# Nutritional Predictors of Mortality in Long Term Hemodialysis Patients

**DOI:** 10.1038/srep35639

**Published:** 2016-10-18

**Authors:** Cheng-Hao Weng, Ching-Chih Hu, Tzung-Hai Yen, Ching-Wei Hsu, Wen-Hung Huang

**Affiliations:** 1 Department of Nephrology and Division of Clinical Toxicology and Toxicology Laboratory, Chang Gung Memorial Hospital, Linkou Medical Center, Taiwan; 2 Chang Gung University College of Medicine, Taoyuan, Taiwan; 3 Department of Hepatogastroenterology and Liver Research Unit, Chang Gung Memorial Hospital, Keelung, Taiwan

## Abstract

Serum albumin had been noted to be a predictor of mortality in hemodialysis (HD) patients. Normalized protein catabolic rate (nPCR) less than 0.8 or greater than 1.4 g/kg/d was also associated with greater mortality. There was no previous study to show the effectiveness of combination of serum albumin and nPCR to predict the mortality in chronic HD patients. Eight hundred and sixty-six patients were divided into 4 groups according to their nPCR and serum albumin levels. Biochemical, and hematological parameters were recorded. The associations between groups, variables mentioned above and mortality were analyzed. Multivariate Cox regression analysis showed that age, diabetes mellitus, fistula as blood access, nPCR <1.2 g/kg/day combined with albumin <4 (Group A), nPCR ≧ 1.2 g/kg/day combined with albumin <4 g/dL (Group B) (nPCR ≧ 1.2 g/kg/day combined with Albumin ≧ 4 g/dL as reference group), non-anuria, hemoglobin, creatinine, and log (high sensitivity C reactive protein) were correlated with 36 months mortality. Group A and group B patients had higher 36 months cardiovascular (CV) and infection related mortality rates as compared with group D patients. In conclusion, Group A and Group B patients had significantly higher rate of all-cause, CV and infection related mortality.

Serum albumin had been noted to be a predictor of mortality in hemodialysis (HD) patients^1^,^2^. In an meta-analysis, Herselman *et al*. also confirmed that there was an inverse relationship between serum albumin level and all-cause and cardiovascular mortality^3^. Normalized protein catabolic rate has also been considered to be an indication of nutritional status in dialysis patients^4^,^5^. Normalized protein catabolic rate less than 0.8 or greater than 1.4 g/kg/d was associated with greater mortality^6^. Combination of serum albumin and normalized protein catabolic rate (nPCR) can predict mortality in incident HD patients^7^. Lukowsky *et al*. showed that low serum albumin and nPCR in the first quarters of dialysis were associated with mortality in the first 24 months after initiation of dialysis of HD patients in United States, where mortality was exceptionally high in the first year of dialysis^8^. However, the cumulative survival rate of was 96.5% at one year in Taiwan^9^. Therefore, we were more interested in the nutritional predictors of mortality of long-term HD patients in Taiwan. Jiang *et al*. had reported that HD patients with lower serum potassium and uric acid had higher long term mortality rated and they also had lower serum albumin and nPCR^10^. There was no previous study to show the effectiveness of combination of serum albumin and nPCR to predict the mortality in chronic HD patients.

## Results

### Subject characteristics

As shown in Table 1, eight hundred and sixty six patients were included. The patients on HD were 56.18 ± 13.59 years old and 440 patients were male (50.8%). The average HD duration was 6.96 ± 5.35 years. There were significant differences in the means of hemoglobin (Hb), nPCR, serum albumin, creatinine (Cr), corrected calcium, phosphate, iPTH, hsCRP, total cholesterol, triglyceride and low density lipoprotein and ages; and frequency of DM, hepatitis C infection (HCV), fistula as blood access, and residual daily urine >100 ml.

### Clinical predictors of 12 months mortality

Univariate cox regression analysis showed that age [odds ratio (OR) = 1.067, 95% confidence interval (CI) = 1.043–1.092, P < 0.001], DM (OR = 2.440 95% CI = 1.402–4.247, P = 0.002), fistula as blood access (OR = 0.472, 95% CI = 0.267–0.837, P = 0.020), nPCR <1.2 g/kg/day combined with albumin <4 g/dL (OR = 7.443, 95% CI = 2.829–19.577, P < 0.001), nPCR ≧ 1.2 g/kg/day combined with albumin <4 g/dL (OR = 6.211, 95% CI = 2.123–18.170, P = 0.001) (nPCR ≧ 1.2 g/kg/day combined with Albumin ≧ 4 g/dL as reference group), non-anuria (OR = 0.150, 95% CI = 0.036–0.614, P = 0.008), white blood cells (WBC) >10000/μL (OR = 4.507, 95% CI = 2.032–9.996, P < 0.001), Hb (OR = 0.628, 95% CI = 0.518–0.761, P < 0.001), Cr (OR = 0.734, 95% CI = 0.649–0.830, P < 0.001), phosphate (OR = 0.669, 95% CI = 0.537–0.834, P < 0.001), Log iPTH (OR = 0.619, 95% CI = 0.418–0.916, P = 0.016), and Log hsCRP (OR = 4.405, 95% CI = 2.539–7.641, P < 0.001) were correlated with 12 months mortality (Table 2). Multivariate Cox regression analysis showed that age (OR = 1.059, 95% CI = 1.033–1.086, P < 0.001), DM (OR = 1.846, 95% CI = 1.039–3.282, P = 0.037), hepatitis B infection (HBV) (OR = 2.197, 95% CI = 1.032–4.679, P = 0.041), non-anuria (OR = 0.222, 95% CI = 0.054–0.919, P = 0.038), Hb (OR = 0.717, 95% CI = 0.587–0.877, P = 0.001), and Log hsCRP (OR = 2.297, 95% CI = 1.271–4.153, P = 0.006) were correlated with 12 months mortality (Table 3).

### Clinical predictors of 24 months mortality

Univariate cox regression analysis showed that age (OR = 1.077, 95% CI = 1.060–1.095, P < 0.001), DM (OR = 3.270 95% CI = 2.224–4.809, P < 0.001), previous CV diseases (OR = 1.978, 95% CI = 0.999–3.919, P = 0.005). HD duration (OR = 0.960 95% CI = 0.922–0.999, P = 0.045), fistula as blood access (OR = 0.437, 95% CI = 0.293–0.653, P < 0.001), hemodiafiltration (OR = 0.551 95% CI = 0.314–0.967, P = 0.038), nPCR <1.2 g/kg/day combined with albumin <4 g/dL (OR = 6.735, 95% CI = 3.473–13.061, P < 0.001), nPCR ≧ 1.2 g/kg/day combined with albumin <4 g/dL (OR = 5.569, 95% CI = 2.650–11.702, P < 0.001), nPCR < 1.2 g/kg/day combined with albumin ≧ 4 g/dL (OR = 2.691, 95% CI = 1.352–5.353, P = 0.005) (nPCR ≧ 1.2 g/kg/day combined with Albumin ≧ 4 g/dL as reference group), non-anuria (OR = 0.305, 95% CI = 0.148–0.627, P = 0.001), WBC > 10000/μL (OR = 2.520, 95% CI = 1.225–5.184, P = 0.012), Hb (OR = 0.750, 95% CI = 0.649–0.866, P < 0.001), serum Cr (OR = 0.709, 95% CI = 0.649–0.775, P < 0.001), phosphate (OR = 0.706, 95% CI = 0.605–0.824, P < 0.001), Log ferritin (OR = 1.892, 95% CI = 1.187–3.017, P = 0.007), Log iPTH (OR = 0.582, 95% CI = 0.422–0.764, P < 0.001), and Log hsCRP (OR = 2.900, 95% CI = 1.976–4.257, P < 0.001) were correlated with 24 months mortality (Table 2). Multivariate Cox regression analysis showed that age (OR = 1.056, 95% CI = 1.036–1.076, P < 0.001), DM (OR = 2.136, 95% CI = 1.413–3.229, P < 0.001), non-anuria (OR = 0.362, 95% CI = 0.168–0.784, P = 0.010), Cr (OR = 0.836, 95% CI = 0.755–0.924, P < 0.001), and log hsCRP (OR = 2.124, 95% CI = 1.430–3.153, P < 0.001) were correlated with 24 months mortality (Table 3).

### Clinical predictors of 36 months mortality

Univariate cox regression analysis showed that age (OR = 1.078, 95% CI = 1.064–1.092, P < 0.001), DM (OR = 2.408, 95% CI = 1.747–3.319, P < 0.001), previous CV diseases (OR = 1.929, 95% CI = 1.094–3.402, P = 0.023). hemodialysis duration (OR = 0.995 95% CI = 0.924–0.986, P = 0.005), fistula as blood access (OR = 0.360, 95% CI = 0.262–0.495, P < 0.001), hemodiafiltration (OR = 0.441 95% CI = 0.270–0.720, P = 0.001), nPCR <1.2 g/kg/day combined with Albumin < 4 (OR = 7.516, 95% CI = 4.420–12.783, P < 0.001), nPCR ≧ 1.2 g/kg/day combined with Albumin <4 g/dL (OR = 5.200, 95% CI = 2.821–9.584, P < 0.001), nPCR < 1.2 g/kg/day combined with Albumin ≧ 4 g/dL (OR = 2.682, 95% CI = 1.540–4.671, P < 0.001) (nPCR ≧ 1.2 g/kg/day combined with Albumin ≧ 4 g/dL as reference group), non-anuria (OR = 0.372, 95% CI = 0.218–0.633, P < 0.001), Hb (OR = 0.726, 95% CI = 0.645–0.871, P < 0.001), serum Cr (OR = 0.720, 95% CI = 0.669–0.774, P < 0.001), phosphate (OR = 0.725, 95% CI = 0.639–0.821, P < 0.001), Log ferritin (OR = 2.404, 95% CI = 1.616–3.576, P < 0.001), Log iPTH (OR = 0.643, 95% CI = 0.511–0.809, P < 0.001), cholesterol (OR = 0.994, 95% CI = 0.989–0.998, P = 0.004) and Log hsCRP (OR = 2.591, 95% CI = 1.902–3.528, P < 0.001) were correlated with 36 months mortality (Table 2). Multivariate Cox regression analysis showed that age (OR = 1.052, 95% CI = 1.036–1.068, P < 0.001), DM (OR = 1.661, 95% CI = 1.78–2.340, P = 0.004), fistula as blood access (OR = 0.620, 95% CI = 0.439–0.874, P = 0.006), nPCR <1.2 g/kg/day combined with albumin <4 g/dL (OR = 2.241, 95% CI = 1.251–4.015, P = 0.007), nPCR ≧ 1.2 g/kg/day combined with albumin <4 g/dL (OR = 2.690, 95% CI = 1.428–5.069, P = 0.002) (nPCR ≧ 1.2 g/kg/day combined with albumin ≧ 4 g/dL as reference group), non-anuria (OR = 0.466, 95% CI = 0.266–0.814, P = 0.007), Hb (OR = 0.872, 95% CI = 0.764–0.994, P = 0.041), Cr (OR = 0.905, 95% CI = 0.827–0.992, P = 0.032), and Log hsCRP (OR = 1.478, 95% CI = 1.058–2.066, P = 0.022) were correlated with 36 months mortality (Table 3).

### Cardiovascular mortality

**Figure 1:**
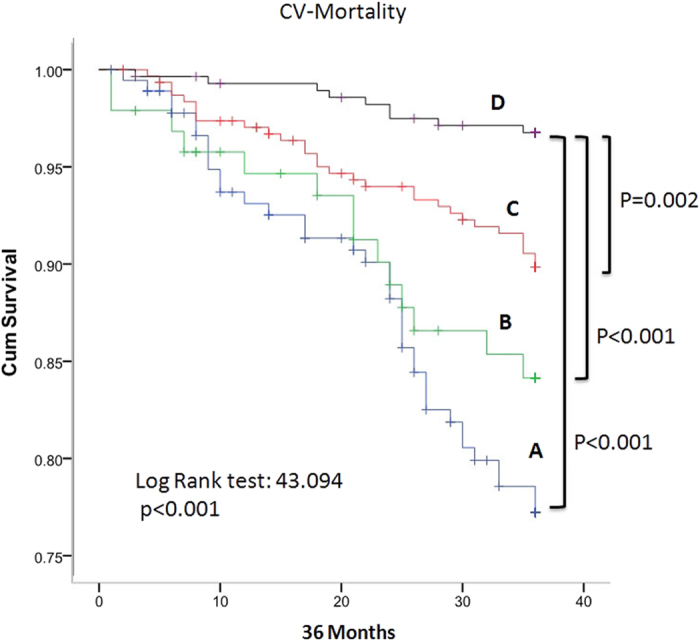
Cumulative survival rates based on cardiovascular mortality and different groups of hemodialysis patients. CV: cardiovascular; (**A–D**) Group (**A–D**) patients.

Cox regression analysis showed that group A patients had significantly highest 36 months CV mortality rate as compared with group D patients (P < 0.001). Group B and group C patients also had higher 36 months CV mortality rates as compared with group D patients (P < 0.001 and P = 0.002, respectively (Fig. 1)).

### Infection related mortality

**Figure 2:**
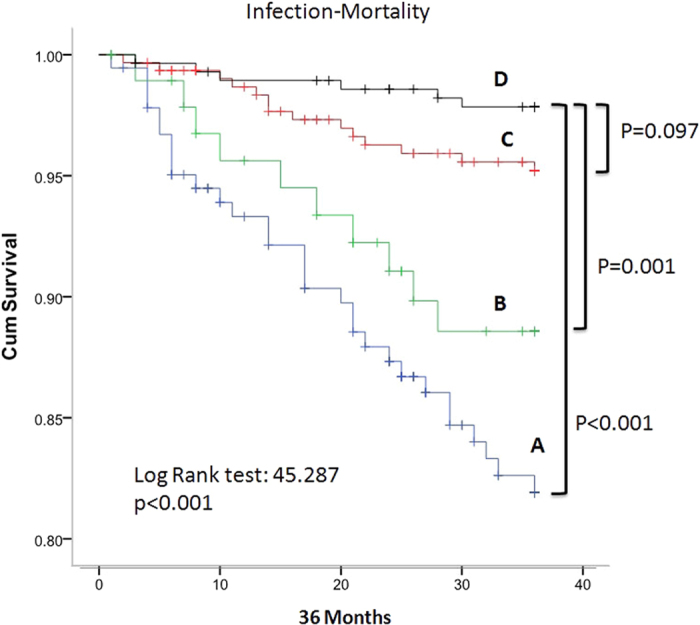
Cumulative survival rates based on infection related mortality and different groups of hemodialysis patients. (**A–D**) Group (**A–D**) patients.

Multivariate Cox regression on infection related 36 months mortality, after adjustment with clinical predictors, including fistula as blood ascess (OR:0.583, 95% CI [0.341–0.996], P = 0.048) showed that group A patients had significantly highest 36 months infection related mortality rate as compared with group D patients (P < 0.001). Group B patients also had higher infection related mortality rate as compared with group D patients (P = 0.001) (Fig. 2).

## Discussion

Lukowsky *et al*.^7^ showed that serum albumin <3.5 g/dL was consistently associated with high mortality as was nPCR <1 g/kg/day in the incident HD patients. Decreasing of serum albumin and nPCR greater than 0.2 g/dL or g/kg/day, respectively, were associated with increased risk of death. Quarterly rise in nPCR (> +0.2 g/kg/day) showed reverse effect on mortality from the 2nd to the last quarter. Our present study showed that long term HD patients with nPCR <1.2 g/kg/day combined with albumin <4 g/dL had significantly higher mortality (nPCR ≧ 1.2 g/kg/day combined with albumin ≧ 4 g/dL as reference group) and they also had significantly higher CV and infection related mortality than other groups. The present K/DOQI clinical practice guideline for hemodialysis^11^ suggested stabilized serum albumin equal to or greater than 4.0 g/dL. Our study using the suggestion by K/DOQI that serum albumin should be >4 g/dL in HD patients and demonstrated that patients on chronic HD with nPCR ≧ 1.2 g/kg/day combined with albumin <4 g/dL (group A) had even higher odds ratio for all-cause mortality than patients with nPCR <1.2 g/kg/day combined with Albumin <4 g/dL (2.690 vs. 2.241) (group B). Group A patients had higher hsCRP than group B patients. Kaysen *et al*.^12^ showed that at progressively greater levels of CRP, serum albumin concentration decreased even if nPCR values were high. Elevated levels of CRP were significantly associated with all-cause mortality in dialysis patients^13^. Normalized protein catabolic rate may overestimate dietary protein intake because of endogenous nitrogen breakdown in the condition of inflammation^14^.

Our study showed that patients with nPCR < 1.2 g/kg/day combined with albumin >4 g/dL (group C) did not have higher odds ratio for all-cause mortality than patients with nPCR > 1.2 g/kg/day combined with albumin >4 (group D, the reference group). Shiniberger *et al*.^6^ had showed that the best survival of HD patients was associated with nPCR between 1.0 and 1.4 g/kg/d, and patients with nPCR less than 0.8 or greater than 1.4 g/kg/d was associated with greater mortality. The K/DOQI guidelines also suggested that nPCR between 1.0 and 1.2 g/kg/d^15^. Although the patients in group C had nPCR < 1.2 g/kg/day, but their average nPCR was 1.01 ± 0.13 g/kg/d, which was in the range by K/DOQI and Shiniberger *et al*. These might explain that group C patients did not have higher overall mortality rate than group D patients.

Our study showed that patients of group A, group B, and group C had significantly higher CV mortality than patients of group D. The baseline characteristic data (Table 1) showed that there were significantly progressive increasing levels of hsCRP from patients of group D to group A. High sensitivity CRP had been a well-known predictor of CV mortality in dialysis patients^16^,^17^,^18^.

Patients of group A and group B had significantly higher infection-related mortality than patients of group D in our study. Infection had been noted to be the second most common etiology of mortality in HD patients^19^. And group A and group B patients had significantly lower percentage of using arteriovenous fistula as HD vascular accesses. Choices for Healthy Outcomes in Caring for ESRD Study had demonstrated that using central venous catheters as HD vascular access had significantly higher mortality than using AVF^20^. Rivara *et al*. also showed that using CVCs was associated with higher mortality and hospitalization in patients on home HD^21^. Central venous catheters were also associated with significantly higher risk of sepsis as compared with AVFs^22^. Lower percentage of using fistula as blood access in group A and B patients would lead to more HD catheter related infections. Hemodialysis catheter infections were also independently associated with lower serum albumin^23^. Hemodialysis catheter infections had been noted to increase infection related mortality in HD patients^24^. The higher infection related mortality in group A and B patients and its correlation with lower serum albumin might be related more HD catheter infections.

### Limitations

There were several limitations in this study. First, this was a retrospective study and the causal and effect relationships between albumin, nPCR and all-cause, CV, and infection related mortality need further prospective study to see whether improvement of albumin and nPCR can improve mortality in HD patients. Second, the patient numbers were different in different serum and nPCR groups and there were only 95 patients in Group B. Small patient number will increase the possibility of type II errors. We should enroll more Group B patients by cooperation with other HD centers to correct this bias.

## Conclusions

Normalized protein catabolic rate ≧1.2 g/kg/day combined with albumin <4 g/dL and nPCR <1.2 g/kg/day combined with Albumin <4 were independent predictors of all cause mortality in chronic HD patients. Patients with nPCR  ≧ 1.2 g/kg/day combined with albumin <4 g/dL and nPCR <1.2 g/kg/day combined with Albumin <4 had higher CV and infection related mortality rate than patients with nPCR ≧ 1.2 g/kg/day combined with albumin >4 g/dL.

## Methods

The Institutional Review Board (IRB) Committee of Chang Gung Memorial Hospital approved the study protocol (Code of IRB: 98-1937B). The methods in the study were carried out in accordance with the approved guidelines. Informed consent was not required in this retrospective study and our IRB committee approved this. Senior nephrologists reviewed all medical records during the study period, including medical history, laboratory data, and inclusion and exclusion factors. In addition, all individual information was securely protected and was only available to the investigators. Finally, all primary data were collected according to the Strengthening the Reporting of Observational Studies in Epidemiology guidelines.

### Patients

Study patients were recruited from the 3 hemodialysis centers of Chang Gung Memorial Hospital, Lin-Kou Medical Center, Taipei and Taoyuan branches. Recruitment started since February 2013, and patients were followed up for 36 months. Only prevalent maintenance hemodialysis (MHD) patients who were 18 years of age or older and had received hemodialysis for at least 6 months were enrolled in this study. Patients with malignancies or obvious infectious diseases, as well as those who had been hospitalized or had undergone surgery within 3 months of the investigation, were excluded. Diabetes mellitus (DM) was defined by either a physician’s diagnosis, anti-diabetic drug treatment, or if 2 subsequent analyses demonstrated fasting blood glucose levels of >126 mg/dl. Patients were defined as having hypertension if they were taking anti-hypertensive drugs regularly or their blood pressure was >140/90 mmHg on at least 2 occasions. Most patients underwent 4 hours of HD, 3 times a week. Hemodialysis was performed using single-use hollow-fiber dialyzers equipped with modified cellulose, polyamide, or polysulfone membranes. The dialysate used in all cases had a standard ionic composition with a bicarbonate-based buffer. We noted the incidence of cardiovascular (CV) diseases including cerebrovascular disease, coronary artery disease, congestive heart failure, and peripheral vascular disease in these patients. The causes of mortality were also recorded. In the analysis of predictors of CV mortality, patients with previous CV disease were not included.

### Laboratory Parameters

All the blood samples were drawn from the arterial end of the vascular access immediately after the initial 2-day interval for HD and were then centrifuged and stored at −80 °C until use.

### Albumin and nPCR as Predictors of Mortality

According to the Kidney Disease Outcomes Quality Initiative (K/DOQI) Clinical Practice Guidelines for Chronic Kidney Disease (CKD), a serum albumin level of ≥4.0 g/dL in MHD patients is acceptable^25^. By Lukowsky *et al*.^7^, patients with a high nPCR and low serum albumin likely have adequate nutrition and inflammation; patients with a low nPCR and adequate serum albumin level may have an inadequate nutritional status but may also be less likely to have inflammation; patients with both a low nPCR and low serum albumin level may be malnourished and have inflammation; and patients with both a high nPCR and high serum albumin level are more likely to have neither of the 2 conditions. Therefore, on the basis of these assumptions and the K/DOQI Clinical Practice Guidelines^25^, we divided the patients according to their nPCR and serum albumin levels into 4 groups: (Group A) nPCR < 1.2 g/kg/day and serum albumin level <4 g/dL; (Group B) nPCR ≥ 1.2 g/kg/day and serum albumin level <4 g/dL; (Group C) nPCR < 1.2 g/kg/day and serum albumin level ≥4 g/dL; and (Group D) nPCR ≥ 1.2 g/kg/day and serum albumin level ≥4 g/dL.

### Statistical Analysis

Data were analyzed using SPSS, version 12.0 for Windows 95 (SPSS Inc, Chicago, IL). The Kolmogorov–Smirnov test was used to test if variables were normally distributed. A P value of >0.05 was required to assume a normal distribution. Unless otherwise stated, continuous variables are expressed as mean ± standard deviation or median (interquartile range), and categorical variables are expressed as numbers or percentages. *X*^*2*^ or Fisher exact tests were used to analyze the correlation between categorical variables. Comparisons between 4 groups were performed using the Kruskal–Wallis test and least significant difference (LSD) one-way analysis of variance (ANOVA). Risk factors for pre-dialysis hypotension were assessed by performing univariate logistic regression analysis, and all variables and variables with P < 0.1 were included in a multivariate analysis by applying a multiple logistic regression based on forward elimination of data, respectively. The data of intact parathyroid hormone (iPTH), serum ferritin, and high sensitivity C reactive protein (hsCRP) levels were log transformed for regression analysis due to wide range of standard deviation^26^. Risk factors for mortality were assessed by univariate Cox regression analysis, and variables with P < 0.1 were included in a multivariate analysis by applying a multiple Cox regression.

## Additional Information

**How to cite this article**: Weng, C.-H. *et al*. Nutritional Predictors of Mortality in Long Term Hemodialysis Patients. *Sci. Rep.* **6**, 35639; doi: 10.1038/srep35639 (2016).

## Figures and Tables

**Table 1:** Baseline characteristics of 866 MHD patients and groups based on nPCR and serum albumin levels.

Characteristics	Total (866) Mean ± SD/Median (Range)	Group A (183) Mean ± SD/Median (Range)	Group B (95) Mean ± SD/Median (Range)	Group C (306) Mean ± SD/Median (Range)	Group D (282) Mean ± SD/Median (Range)	P
*Demographics*						
Age (y)	56.18 ± 13.59	63.55 ± 13.57#	58.99 ± 11.25#	54.83 ± 13.98#	51.93 ± 11.67	<0.001
Male sex	440 (50.8%)	90 (49.2%)	32 (33.7%)	185 (60.5%)	133 (47.2%)	0.46
Body mass index (kg/m^2^)	22.19 ± 3.18	22.11 ± 3.35	22.05 ± 3.16	22.23 ± 3.18	22.24 ± 3.09	0.93
Smoking (Yes)	150 (17.3%)	34 (18.6%)	8 (8.4%)	71 (23.2%)	37 (13.1%)	0.51
*Co-Morbidity*						
Diabetes mellitus (Yes)	192 (22.2%)	60 (32.8%)	16 (16.8%)	73 (23.9%)	43 (15.2%)	<0.001
Hypertension (Yes)	339 (39.1%)	69 (37.7%)	32 (33.7%)	134 (43.8%)	104 (36.9%)	0.79
Previous CVD (Yes)	41 (4.7%)	12 (6.6%)	3 (3.2%)	19 (6.2%)	7 (2.5%)	0.098
HBV (Yes)	98 (11.3%)	27 (14.8%)	9 (9.5%)	36 (11.8%)	26 (9.2%)	0.1
HCV (Yes)	168 (19.4%)	45 (24.6%)	33 (34.7%)	50 (16.3%)	40 (14.2%)	<0.001
*Dialysis Related Data*						
Haemodialysis duration (y)	6.96 ± 5.35	7.1 ± 6.0	8.9 ± 5.6#	6.28 ± 5.06	6.93 ± 4.97	<0.001
Erythropoietin (U/kg/week)	73.62 ± 47.37	70.72 ± 49.67	80.03 ± 55.16	72.79 ± 46.50	74.23 ± 43.85	0.46
Fistula as blood access (Yes)	689 (79.6%)	126 (68.9%)	74 (77.9%)	248 (81.0%)	241 (85.5%)	<0.001
Hemodiafiltration (Yes)	187 (21.6%)	31 (16.9%)	29 (30.5%)	64 (20.9%)	63 (22.3%)	0.42
Kt/V Daugirdes	1.79 ± 0.32	1.78 ± 0.33	1.92 ± 0.35#	1.73 ± 0.3#	1.83 ± 0.32	<0.001
nPCR (g/kg/day)	1.18 ± 0.26	0.97 ± 0.16#	1.45 ± 0.20	1.01 ± 0.13#	1.42 ± 0.17	<0.001
Residual daily urine of >100 ml	178 (20.6%)	18 (9.8%)	14 (14.7%)	78 (25.5%)	68 (24.1%)	<0.001
*Biochemical Data*						
Haemoglobin (g/dl)	10.51 ± 1.36	10.28 ± 1.54#	10.24 ± 1.43#	10.59 ± 1.39	10.66 ± 1.15	0.004
Albumin (g/dl)	4.06 ± 0.34	3.65 ± 0.23#	3.7 ± 0.2#	4.24 ± 0.21	4.25 ± 0.21	<0.001
Creatinine (mg/dl)	10.88 ± 2.39	9.45 ± 2.4#	10.54 ± 2.11#	11.13 ± 2.34#	11.66 ± 2.08	<0.001
Corrected-calcium (mg/dl)	9.94 ± 0.93	9.65 ± 1.0#	9.96 ± 1.09	9.97 ± 0.86	10.09 ± 0.85	<0.001
Phosphate (mg/dl)	4.84 ± 1.35	4.21 ± 1.35#	4.94 ± 1.12#	4.77 ± 1.29#	5.29 ± 1.32	<0.001
Ferritin (μg/l)#	305.0 (129.57, 504.45)	285.3 (118.85, 487.75)	298 (114.8, 491.15)	292.9 (138, 495.8)	332.1 (130, 522.2)	0.60
Intact parathyroid hormone (pg/ml)#	130.1 (52.52, 319.2)	94.7 (34.8, 239.7)#	202.6 (58, 431.9)	129.75 (52.37, 293.82)	146.1 (61.15, 356.95)	<0.001
hsCRP (mg/l)#	2.95 (1.4, 7.01)	4.95 (2.17, 11.27)#	4.32 (1.9, 10.2)#	2.66 (1.35, 6.01)	2.2 (1.12, 4.79)	<0.001
*Cardiovascular Risks*						
Cholesterol (mg/dl)	171.3 ± 37.66	159.6 ± 38.92#	174.89 ± 41.61	166.46 ± 32.52#	182.94 ± 37.44	<0.001
Triglyceride (mg/dl)	164.33 ± 115.8	149.98 ± 94.13#	154.39 ± 124.71	163.11 ± 118.59	178.3 ± 121.28	0.054
LDL (mg/dl)	94.83 ± 30.59	89.37 ± 31.87#	98.89 ± 34.12	90.23 ± 26.5#	101.89 ± 31.19	<0.001

^#^p < 0.05 by comparing with group D.

Group A: nPCR<1.2 g/kg/day combined with Albumin <4 g/dL; Group B: nPCR ≧ 1.2 g/kg/day combined with Albumin <4 g/dL; Group C: nPCR < 1.2 g/kg/day combined with Albumin ≧ 4 g/dL; Group D: nPCR ≧ 1.2 g/kg/day combined with Albumin ≧ 4 g/dL.

nPCR: normalized protein catabolic rate. CVD: cardiovascular diseases included cerebrovascular disease, coronary arterial disease, congestive heart failure, and peripheral vascular disease. HDF: hemodiafiltration, HBV: hepatitis B infection, HCV: hepatitis C infection, hsCRP: high sensitivity C reactive protein, LDL: low density lipoprotein.

*Non-normal distribution data are presented as median (interquartile range).

**Table 2:** Univariate Cox Regression Analysis of 12-, 24-, and 36-month all-cause mortality.

Characteristics	Univariate Cox regression 12 months	p	Univariate Cox regression 24 months	p	Univariate Cox regression 36 months	p
*Variables*	Odds ratio (OR) 95% confidence Intervals (CI)		Odds ratio (OR) 95% confidence Intervals (CI)		Odds ratio (OR) 95% confidence Intervals (CI)	
Age (years)	1.067 (1.043–1.092)	0.000	1.077 (1.060–1.095)	0.000	1.078 (1.064–1.092)	0.000
Male sex	0.825 (0.478–1.423)	0.489	0.758 (0.515–1.116)	0.160	0.870 (0.637–1.187)	0.379
Body mass index (kg/m^2^)	1.066 (0.985–1.153)	0.114	1.025 (0.965–1.087)	0.423	1.016 (0.968–1.067)	0.512
Smoking (Yes)	0.851 (0.400–1.807)	0.674	0.919 (0.546–1.545)	0.749	1.103 (0.741–1.640)	0.630
Diabetes mellitus (Yes)	2.440 (1.402–4.247)	0.002	3.270 (2.224–4.809)	0.000	2.408 (1.747–3.319)	0.000
Hypertension (Yes)	1.151 (0.664–1.995)	0.617	1.021 (0.689–1.513)	0.916	0.950 (0.689–1.309)	0.752
Previous CVD (Yes)	1.673 (0.603–4.641)	0.323	1.978 (0.999–3.919)	0.050	1.929 (1.094–3.402)	0.023
HBV (Yes)	1.904 (0.955–3.794)	0.067	1.149 (0.643–2.055)	0.638	1.020 (0.624–1.665)	0.938
HCV (Yes)	0.982 (0.493–1.958)	0.960	0.925 (0.562–1.520)	0.757	0.921 (0.616–1.377)	0.689
Hemodialysis duration (years)	0.980 (0.929–1.034)	0.459	0.960 (0.922–0.999)	0.045	0.955 (0.924–0.986)	0.005
Fistula as blood access (Yes)	0.472 (0.267–0.837)	0.010	0.437 (0.293–0.653)	0.000	0.360 (0.262–0.495)	0.000
Hemodiafiltration (Yes)	0.753 (0.367–1.545)	0.439	0.551 (0.314–0.967)	0.038	0.441 (0.270–0.720)	0.001
Kt/V_urea_ (Daugirdes)	0.709 (0.303–1.660)	0.428	0.751 (0.412–1.367)	0.349	0.673 (0.412–1.099)	0.114
*nPCR* (*g/kg/day*) *combined with albuimn* (*g/dL*)		<0.001		<0.001		<0.001
nPCR <1.2 combined with albumin <4	7.443 (2.829–19.577)	0.000	6.735 (3.473–13.061)	0.000	7.516 (4.420–12.783)	0.000
nPCR ≧ 1.2 combined with albumin <4	6.211 (2.123–18.170)	0.001	5.569 (2.650–11.702)	0.000	5.200 (2.821–9.584)	0.000
nPCR<1.2 combined with albumin ≧ 4	2.613 (0.941–7.256)	0.065	2.691 (1.352–5.353)	0.005	2.682 (1.540–4.671)	0.000
nPCR ≧ 1.2 combined with albumin ≧ 4 (reference)						
Non-Anuria	0.150 (0.036–0.614)	0.008	0.305 (0.148–0.627)	0.001	0.372 (0.218–0.633)	0.000
Hemoglobin (g/dl)	0.628 (0.518–0.761)	0.000	0.750 (0.649–0.866)	0.000	0.726 (0.645–0.817)	0.000
WBC >10000/μL	4.507 (2.032–9.996)	0.000	2.520 (1.225–5.184)	0.012	1.839 (0.938–3.604)	0.076
Creatinine (mg/dl)	0.734 (0.649–0.830)	0.000	0.709 (0.649–0.775)	0.000	0.720 (0.669–0.774)	0.000
C-Ca	1.037 (0.776–1.385)	0.806	0.900 (0.730–1.111)	0.327	0.908 (0.766–1.076)	0.264
Phosphate (mg/dl)	0.669 (0.537–0.834)	0.000	0.706 (0.605–0.824)	0.000	0.725 (0.639–0.821)	0.000
Log ferritin	1.518 (0.805–2.863)	0.197	1.892 (1.187–3.017)	0.007	2.404 (1.616–3.576)	0.000
Log PTH	0.619 (0.418–0.916)	0.016	0.582 (0.442–0.764)	0.000	0.643 (0.511–0.809)	0.000
Log hsCRP	4.405 (2.539–7.641)	0.000	2.900 (1.976–4.257)	0.000	2.591 (1.902–3.528)	0.000
Cholesterol (mg/dl)	0.998 (0.990–1.005)	0.508	0.996 (0.990–1.001)	0.105	0.994 (0.989–0.998)	0.004
Triglyceride (mg/dl)	1.000 (0.998–1.003)	0.724	1.000 (0.999–1.002)	0.667	1.000 (0.999–1.001)	0.857

MHD: maintenance haemodialysis, CVD: cardiovascular disease, nPCR: normalized protein catabolic rate, HBV: hepatitis B virus infection, HCV: hepatitis C virus infection, DM = diabetes mellitus, hsCRP = high sensitivity C reactive protein, iPTH = intact parathyroid hormone, Kt/V urea = dialysis clearance of urea, C-Ca = corrected calcium.

**Table 3:** Multivariate Cox Regression Analysis of 12-, 24-, and 36-month all-cause mortality.

Characteristics	Multivariate Cox regression 12 months	p	Multivariate Cox regression 24 months	p	Multivariate Cox regression 36 months	p
*Variables*	Odds ratio (OR) 95% confidence Intervals (CI)		Odds ratio (OR) 95% confidence Intervals (CI)		Odds ratio (OR) 95% confidence Intervals (CI)	
Age (years)	1.059 (1.033–1.086)	0.000	1.056 (1.036–1.076)	0.000	1.052 (1.036–1.068)	0.000
Male sex						
Body mass index (kg/m^2^)						
Smoking (Yes)						
Diabetes mellitus (Yes)	1.846 (1.039–3.282)	0.037	2.136 (1.413–3.229)	0.000	1.661 (1.178–2.340)	0.004
Hypertension (Yes)						
Previous CVD (Yes)						
HBV (Yes)	2.197 (1.032–4.679)	0.041				
HCV (Yes)						
Hemodialysis duration (years)						
Fistula as blood access (Yes)					0.620 (0.439–0.874)	0.006
Hemodiafiltration (Yes)						
Kt/V_urea_ (Daugirdes)						
*nPCR* (*g/kg/day*) *combined with albuimn* (*g/dL*)						0.013
nPCR <1.2 combined with albumin <4					2.241 (1.251–4.015)	0.007
nPCR ≧ 1.2 combined with albumin <4					2.690 (1.428–5.069)	0.002
nPCR<1.2 combined with albumin ≧ 4						
nPCR ≧ 1.2 combined with albumin ≧ 4 (reference)						
Non-Anuria	0.222 (0.054–0.919)	0.038	0.362 (0.168–0.784)	0.010	0.466 (0.266–0.814)	0.007
Hemoglobin (g/dL)	0.717 (0.587–0.877)	0.001			0.872 (0.764–0.994)	0.041
WBC >10000/μL	2.374 (0.992–5.682)	0.052				
Creatinine (mg/dL)			0.836 (0.755–0.924)	0.000	0.905 (0.827–0.992)	0.032
C-Ca						
Phosphate (mg/dL)						
Log ferritin						
Log PTH						
Log hsCRP	2.297 (1.271–4.153)	0.006	2.124 (1.430–3.153)	0.000	1.478 (1.058–2.066)	0.022
Cholesterol (mg/dL)						
Triglyceride (mg/dL)						

MHD: maintenance haemodialysis, CVD: cardiovascular disease, nPCR: normalized protein catabolic rate, HBV: hepatitis B virus infection, HCV: hepatitis C virus infection, DM = diabetes mellitus, hsCRP = high sensitivity C reactive protein, iPTH = intact parathyroid hormone, Kt/V urea = dialysis clearance of urea, C-Ca = corrected calcium.
